# Mature primary human osteocytes in mini organotypic cultures secrete FGF23 and PTH1-34-regulated sclerostin

**DOI:** 10.3389/fendo.2023.1167734

**Published:** 2023-05-08

**Authors:** Helen J. Knowles, Anastasios Chanalaris, Argyro Koutsikouni, Adam P. Cribbs, Liam M. Grover, Philippa A. Hulley

**Affiliations:** ^1^ Botnar Institute for Musculoskeletal Sciences, Nuffield Department of Orthopaedics Rheumatology and Musculoskeletal Sciences, University of Oxford, Oxford, United Kingdom; ^2^ School of Biology, Aristotle University of Thessaloniki, Thessaloniki, Greece; ^3^ Oxford Centre for Translational Myeloma Research, Botnar Institute for Musculoskeletal Sciences, University of Oxford, Oxford, United Kingdom; ^4^ Healthcare Technologies Institute, School of Chemical Engineering, University of Birmingham, Birmingham, United Kingdom

**Keywords:** osteocyte, sclerostin, FGF23, mineralization, osteoclast, organoid

## Abstract

**Introduction:**

For decades, functional primary human osteocyte cultures have been crucially needed for understanding their role in bone anabolic processes and in endocrine phosphate regulation via the bone-kidney axis. Mature osteocyte proteins (sclerostin, DMP1, Phex and FGF23) play a key role in various systemic diseases and are targeted by successful bone anabolic drugs (anti-sclerostin antibody and teriparatide (PTH1-34)). However, cell lines available to study osteocytes produce very little sclerostin and low levels of mature osteocyte markers. We have developed a primary human 3D organotypic culture system that replicates the formation of mature osteocytes in bone.

**Methods:**

Primary human osteoblasts were seeded in a fibrinogen / thrombin gel around 3D-printed hanging posts. Following contraction of the gel around the posts, cells were cultured in osteogenic media and conditioned media was collected for analysis of secreted markers of osteocyte formation.

**Results:**

The organoids were viable for at least 6 months, allowing co-culture with different cell types and testing of bone anabolic drugs. Bulk RNAseq data displayed the developing marker trajectory of ossification and human primary osteocyte formation *in vitro* over an initial 8- week period. Vitamin D3 supplementation increased mineralization and sclerostin secretion, while hypoxia and PTH1-34 modulated sclerostin. Our culture system also secreted FGF23, enabling the future development of a bone-kidney-parathyroid-vascular multi-organoid or organ-on-a-chip system to study disease processes and drug effects using purely human cells.

**Discussion:**

This 3D organotypic culture system provides a stable, long-lived, and regulated population of mature human primary osteocytes for a variety of research applications.

## 1 Introduction

Osteocytes are the most abundant cells in bone and are essential for skeletal function. They reside deep within mineralized bone from where they regulate bone acquisition during skeletal growth and the healthy maintenance of the skeleton throughout life. They act by co-ordinating osteoblast-mediated bone formation and osteoclast-driven bone resorption, by secreting factors that allow skeletal adaptation to mechanical and hormonal stimuli. Osteocytes also contribute to the endocrine functions of bone by secreting hormones that regulate processes including mineral homeostasis and phosphate metabolism (1).

Osteocytes differentiate from mature osteoblasts, the differentiation and survival of both being regulated by Wnt/β-catenin signaling pathways. Early osteocytes first become surrounded by unmineralized matrix and then by mineralized bone as they sequentially express proteins related to dendritic morphology and canaliculi formation (e.g. MMP14, podoplanin), matrix mineralization (e.g. PHEX, DMP-1, MEPE), bone resorption (e.g. RANKL, M-CSF, OPG), inhibition of bone formation (e.g. SOST, PTHR), and phosphate metabolism (e.g. FGF23, PHEX, DMP1) (1).

Interest in these highly specialized cells was elevated following the discovery that mutations in sclerostin (SOST), a protein expressed almost exclusively by mature osteocytes, cause the rare skeletal bone overgrowth conditions van Buchems disease and sclerosteosis (2). Dysregulation of sclerostin has since been implicated in osteoporosis, osteogenesis imperfecta, vascular calcification and breast cancer while other osteocytic genes play functional roles in the pathogenesis of conditions such as hypophosphataemic rickets (DMP1, PHEX, FGF23), chronic kidney disease (FGF23) and tumor-induced osteomalacia (FGF23) (3). Antibody therapies targeting the mature osteocyte markers sclerostin and fibroblast growth factor 23 (FGF23) have emerged as therapeutic strategies to treat these conditions (4, 5).

Despite these indications, knowledge of osteocyte molecular mechanisms relevant to health and disease lags behind that of other cell types due to lack of suitable *in vitro* research tools. The most widely used osteocytic cell line, murine MLO-Y4 cells, expresses early osteocyte markers but only very low levels of mature osteocyte markers such as sclerostin and FGF23. More recently developed murine cell lines, including IDG-SW3 and Ocy454 cells (which exhibit a temperature-sensitive non-proliferative phenotype) and OmGFP66 and OmGFP10 cells (in which membrane-targeted GFP is driven by the Dmp1 promoter) differentiate into mature osteocytes expressing SOST and other osteocyte markers, are responsive to parathyroid hormone (PTH) and mechanical forces, and form 3D structures with osteocytes buried in mineralized matrix (6–8).

While murine cell lines have advanced our understanding of osteocyte biology, they still have significant limitations. These include: (i) continued proliferation in contrast to the post-mitotic nature of osteocytes themselves; (ii) non-physiological close contact of osteocytes in culture versus restricted contact via dendrites of osteocytes confined to their lacunae *in vivo* and (iii) monolayer culture that lacks the mechanosensitivity of osteocytes embedded in a heavily mineralized extracellular matrix (9, 10).

However, the inability to generate human osteocytes *in vitro* is the primary restriction on both our understanding of osteocyte regulatory mechanisms and our ability to screen for osteocyte-targeting drugs. To translate basic discoveries into therapeutics it is important to validate them in human cells before clinical trials. There is therefore an urgent need to develop novel human 3D osteocyte models derived from patient material, which will provide the required platform to translate basic research into clinically relevant and effective treatments.

Human bone-derived cells grown in monolayer culture have a limited capacity to form osteocyte-like cells expressing DMP1 and sclerostin when cultured in mineralization medium supplemented with either strontium ranelate or vitamin K (11, 12). More robust osteocyte formation has been achieved when such cells are seeded onto 3D structured scaffolds made from materials including collagen, hydroxyapatite, calcium phosphate and polystyrene (9, 10). Although such engineered 3D scaffolds only partially replicate the complexity of bone tissue, they do appear to simulate the physiological responsiveness of osteocytes to mechanical loading. Human primary osteocytes differentiated on calcium phosphate microbeads produce an osteoid matrix with osteocyte-like cells individually embedded in lacunae and expressing osteocytic markers including DMP1, Phex, SOST, MEPE, FGF23 and RANKL (13, 14). These cells replicate the mechanotransduction function of osteocytes *in vivo* by down-regulating SOST expression in response to cyclic mechanical loading (14).

Bone itself, in the form of *ex vivo* organ culture, would have the additional advantage of maintaining osteocytes in their native complex matrix environment. Human bone chips release sclerostin and FGF23 from embedded osteocytes (15, 16). However, osteocyte viability is reduced to 60% after only 7 days of culture (16), although this effect can be partially reversed by mechanical loading of the tissue (17). Decellularized femoral head micro-trabeculae have been used as scaffolds and seeded with primary human osteoblastic cells which develop early osteocytic markers and show sensitivity to fluctuations in gravitational forces (18).

This study describes the development of a self-assembling, self-sustaining organotypic model of human bone containing mature, sclerostin- and FGF23-producing primary osteocytes within a mineralized matrix. It is demonstrated that these organoids allow screening for osteocyte-targeting agents via effects on secretion of sclerostin and, as such, represent a valuable new tool for the detailed *in vitro* study of mechanisms of action relevant to osteocyte mechanisms of disease.

## 2 Materials and methods

### 2.1 Materials and ethics

Elephant dentine was obtained from HM Revenue & Customs (Heathrow Airport, UK). Unless stated, other reagents were from Merck Life Science (Gillingham, UK). The use of leucocyte cones for osteoclast differentiation was approved by the London–Fulham Research Ethics Committee (11/H0711/7). Human osteoblasts were obtained by passage from surgical waste tissue obtained either pre-2009 from patients after informed consent and ethical permission was obtained for participation in this study (National Research Ethics Committee Oxfordshire, REC reference number C01.070) or 2009 onwards from the Oxford Musculoskeletal Biobank with informed donor consent in full compliance with national and institutional ethical requirements, the United Kingdom Human Tissue Act, and the Declaration of Helsinki (HTA Licence 12217 and Oxford REC C 09/H0606/11).

### 2.2 Primary human osteoblasts

Bone fragments were obtained from the trabecular bone of patients undergoing joint replacement for osteoarthritis. Bone fragments (approx. 1 mm^2^) were washed in PBS then incubated in 1 mg/ml collagenase at 37°C for 30 min before incubation in a 10 cm dish in osteoblast media (**Table 1**) in a humidified incubator at 37°C, 5% CO_2_. After 2 days, media was replaced and additionally supplemented with 5 μg/ml L-ascorbic acid 2-phosphate. Supplemented media was replaced every 3-4 days and outgrowth osteoblasts were banked in liquid nitrogen after one or more passages, once confluence was reached. Osteoblasts were used for osteocyte constructs up to passage 5. Passaging and dissociation for seeding of osteocyte constructs was performed using TrypLE Select (ThermoFisher Scientific).

**Table 1:** Details of culture media.

Medium	Components
Osteoblast medium	α-MEM 10% FBS 2 mM Glutamax 50 IU/ml penicillin 50 μg/ml streptomycin sulphate
Proliferation medium	osteoblast medium plus 50 μg/ml L-ascorbic acid 2-phosphate 40 μg/ml proline
Osteogenic medium	proliferation medium plus 10 mM β-glycerophosphate 10 nM dexamethasone

### 2.3 Human primary osteocyte constructs

#### 2.3.1 Initial 6-well method

In the first tranche of human constructs for RNAseq experiments, osteoblasts from 4 donors were seeded onto 20 constructs exactly as we described for rat cells (19). Briefly, calcium phosphate (brushite/β-TCP) posts were pinned into a Sylgard 184 silicone elastomer (base and curing agent, Dow Corning Corporation) base layer in 6-well dishes. Cells were seeded in fibrinogen onto a thrombin gel and subsequently cultured in 3ml of proliferation media (**Table 1**) per well until cells had fully contracted the gel between the support posts. Week 0 constructs were harvested and snap-frozen at this point with the remainder transferred to osteogenic media (**Table 1**) and harvested at 2, 4, 6 and 8 weeks. RNAseq data is shown for the 0- and 8-week groups, when osteocyte markers DMP1 and SOST first appeared.

#### 2.3.2 Modified 12-well method

Triplicate 3D-printed scaffolds were designed in-house and printed by 3D LifePrints (Oxford, UK). Seeding of osteocyte constructs was a modification of our method for culture of primary rat osteocytes (19). Sylgard was prepared and 800 μl used to coat the wells of a 12-well plate. After 4 days curing at room temperature, Sylgard-coated wells and scaffolds were sterilized in 70% ethanol, air-dried, and scaffolds were inserted into the Sylgard wells. Each well was coated with 314 μl of thrombin solution (10 U/ml thrombin, 20 μg/ml aprotinin, 400 μM aminohexanoic acid in osteoblast media) before additional mixing of 126 μl of 20 mg/ml fibrinogen and partial setting at 37°C for 30 min. Primary human osteoblasts (2 x 10^5^ cells in 1 ml osteoblast media) were then added on top of the gel and incubated for 1 h before addition of a further 1 ml of media. Cells were subsequently supplemented with proliferation media twice weekly until the cells/gel had contracted entirely between the posts (approx. 2-4 weeks). After this point, constructs were supplemented twice weekly with osteogenic media for up to 6 months, with additional supplementation with 1 μM vitamin D3 (25(OH)D3) or 5 μM FG4592 (Selleckchem, Houston, TX, USA) as required. The ability of cells to contract the gel between the scaffold posts was quantified in ImageJ as ‘area of osteocyte construct/area of well’ from images taken with a portable camera.

### 2.4 RNA sequencing

Constructs were snap frozen in liquid nitrogen and stored at -80°C. RNA was extracted using Trizol in a Cryo-Cup grinder (BioSpec products, Bartlesville, UK) on dry ice. Total RNA was prepared with the Direct-Zol RNA miniprep kit and RNA clean and concentrator (Zymo Research, Cambridge, UK) according to the manufacturer’s instructions. Libraries were prepared using the NEBNext Poly(A) mRNA magnetic isolation module (New England Biosystems, Ipswich, UK) and then the NEBNext ultra-directional RNA library prep kit was used to create the final library. The sample was sequenced using 41bp paired end configuration with an Illumina NextSeq 500 sequencing device. Reads were trimmed using trimmomatic (20), pseaudoaligned using kallisto (21) with an index built from the hg38 cDNA fasta reference sequence, and then quality of the pseudoalignment was assessed using FastQC. Differential gene expression was performed using the sleuth package. Genes were considered differentially regulated based on an adjusted p value < 0.01. Differentially expressed genes between two groups (constructs at 0 weeks and after 8 weeks of treatment with osteogenic medium) were analyzed for enriched terms using GSEA in R. Heatmaps were generated in R using ggplot2. GO and KEGG (GSEA) analysis was performed on WebGestalt.

### 2.5 Viability assays

#### 2.5.1 Secreted LDH

Conditioned media from osteocyte constructs was diluted 1:100 in LDH Storage Buffer (200mM Tris-HCl (pH 7.3), 10% glycerol, 1% BSA), mixed with an equal volume of LDH Detection Reagent and luminescence was measured after 45 min, according to the manufacturer’s instructions (LDH-Glo Cytotoxicity Assay; Promega).

#### 2.5.2 Live/dead stain

Osteocyte constructs were stained with a mixture of calcein AM (live stain) and ethidium homodimer-1 (dead stain) using the LIVE/DEAD Viability/Cytotoxicity Kit (L-3224; Molecular Probes). Images were obtained using a Nikon Eclipse TE300 microscope with an QImaging Retig 2000R Fast 1394 camera (Teledyne Photometrics, Birmingham, UK) and ImagePro Plus software (Media Cybernetics, Rockville, Maryland, USA).

#### 2.5.3 Alamar blue

Mitochondrial dehydrogenase activity was assessed by exposure to 10% Alamar blue (AbD Serotec) for 3 h, with fluorescence read at ex 540 nm/em 590 nm.

### 2.6 MicroCT

Mineralization was analyzed using a SkyScan 1172 microCT scanner (SkyScan, Kontich, Belgium). Constructs were fixed in 10% formalin and then wrapped in wet absorbent paper to prevent drying during scanning. The constructs were scanned at an isotropic pixel size of 10 μm, a voltage of 37 kV and a current of 228 mA, with a 0.5 mm Al filter. Images were reconstructed with NRecon software (SkyScan 1172). To avoid the high variability in the amount of mineralization that occurred immediately around the scaffold posts, bone volume (BV) was calculated using Skyscan CT-Analyzer software for a tissue volume of interest of 300 sections around the mid-point of the constructs (threshold 80). Images of the constructs were produced in Skyscan CT-Volume software.

### 2.7 Histology and immunofluorescent staining

Following microCT, constructs were decalcified in 10% EDTA prior to wax embedding. Tissue sections were baked at 60°C for 1 hour and heat-mediated antigen retrieval at high pH was performed using an automated PT Link (Dako). Samples were blocked in 5% normal goat serum, incubated overnight at 4°C in anti-SOST rabbit polyclonal antibody (ab85799, 22.5 μg/ml; Abcam, Cambridge, UK) and then with goat anti-rabbit IgG (H+L) Alexa Fluor 633 secondary antibody (1:200, ThermoFisher Scientific). Nuclei were visualized with POPO-1 (P3580; Life Technologies) and autofluorescence was blocked with 0.1% Sudan Black B. Rabbit IgG was used as a negative control. Sections were mounted with Vectashield soft mounting medium (H1000). Imaging was performed on a Zeiss LSM 880 confocal microscope with ZEN Black software.

### 2.8 Osteoblast characteristics in monolayer culture

#### 2.8.1 Proliferation

Cells were stained with crystal violet, which stains nuclei independently of cellular metabolic status. Formalin-fixed cells were incubated with 1% crystal violet for 60 minutes at 37°C, dye was extracted with 0.2% Triton X-100 and absorbance read at 550nm.

#### 2.8.2 Osteoblast differentiation (Alkaline Phosphatase Assay)

Cells were lysed in RIPA buffer with protease inhibitors and the lysate was reacted with 4-methyl umbelliferyl phosphate disodium salt for 30 min at 37°C. The reaction was stopped with Na_2_CO_3_ and fluorescence measured at excitation 360 nm/emission 450 nm.

#### 2.8.3 Mineralization

Formalin-fixed cells were stained with 1.5% alizarin red dye solution (pH 4.1; ScienCell, Carlsbad, CA, USA), washed and air-dried. Dye was extracted in 10% acetic acid, neutralized with 10% ammonium hydroxide and absorbance measured at 550 nm.

### 2.9 ELISAs and western blotting

Human DuoSet ELISA kits against SOST (DY1406), FGF23 (DY2604), VEGF (DY293B), RANKL (DY626) and OPG (DY805) were used according to the manufacturer’s instructions (R&D Systems).

Cells were sonicated in lysis buffer (6.2 M urea, 10% glycerol, 5 mM dithiothreitol, 1% sodium dodecyl sulphate, protease inhibitors) before cell extract was separated by 8% SDS-PAGE and transferred onto a PVDF membrane. Membranes were incubated overnight with primary antibodies specific HIF-1α (clone 54, 1:1000; BD Biosciences, Oxford, UK), GLUT1 (ab14683, 1:2500; Abcam, Cambridge, UK) or β-tubulin (clone TUB2.1, 1:2500, Sigma-Aldrich, Dorset, UK). Chemiluminescence was detected using a UVITEC Alliance Q9 gel doc system and densitometry was performed in ImageJ, normalizing experimental bands to the corresponding β-tubulin control.

### 2.10 Osteoclast differentiation and co-culture with osteocyte constructs

Leucocyte cones were obtained from anonymous donors (NHS Blood and Transplant). CD14+ monocytes positively selected from the PBMC fraction (magnetic CD14+ microbeads; Miltenyi Biotech) were seeded at 2 × 10^6^ cells/well in 12-well plates also containing 4 mm diameter dentine discs or glass coverslips. Cells were allowed to adhere overnight in α-MEM containing 10% FBS, 2 mM L-glutamine, 50 IU/ml penicillin and 50 μg/ml streptomycin sulphate.

Dexamethasone was removed from the osteogenic media of osteocyte constructs 1 week prior to co-culture, as this was found to inhibit osteoclast differentiation. Constructs were moved above adherent monocytes and osteoclastogenesis was induced by supplementation with 25 ng/ml M-CSF (R&D Systems, Abingdon, UK) and 50 ng/ml RANKL (Peprotech, London UK) in α-MEM that also contained ascorbate, proline and β-glycerophosphate (osteogenic media without dexamethasone). Media and supplements were replenished every 3-4 days for 10 days.

### 2.11 Osteoclast assays

#### 2.11.1 Tartrate-resistant acid phosphatase staining

Osteoclasts were fixed in formalin. Equal volumes of solution A (10 mg naphthol AS-BI phosphate, 0.5 ml DMSO in 15 ml acetate-tartrate solution [0.2 M acetic acid, 0.2 M sodium acetate, 10 mM sodium tartrate, pH5]) and solution B (20 mg fast violet B salt, 0.5 ml DMSO in 15ml acetate tartrate solution) were incubated on cells for 3 h at 37°C, then washed and air dried. Photographs were obtained on a Nikon Eclipse TE300 microscope with an Axiocam 105 camera (Carl Zeiss AG) and ZEN acquisition software (blue edition; Zeiss). Multi-nucleated cells with three or more nuclei were considered osteoclasts.

#### 2.11.2 Resorption

Osteoclasts were removed from dentine discs by sonication. Resorption tracks were visualized with 0.5% toluidine blue in boric acid. Photographs were obtained on an Olympus BX40 microscope with ZEN acquisition software (blue edition; Zeiss).

### 2.12 Statistics

For graphical data, the number of experimental repeats is represented by the number of data points with error bars indicating standard deviation. Data was analyzed using GraphPad Prism (GraphPad Software, La Jolla, CA, USA). Normality tests were D’Agistono Pearson or Shapiro–Wilk, depending on the sample size. Statistical analysis comprised one-way or two-way ANOVA using Dunnett’s or Tukey’s multiple comparison or Kruskal–Wallis ANOVA with Dunn’s multiple comparison. For experiments with only two conditions, a T test or Mann–Whitney test was applied. Results were considered significant at p < 0.05.

## 3 Results

### 3.1 Human organotypic bone constructs express osteocyte marker mRNAs at 8 weeks

**Figure 1:**
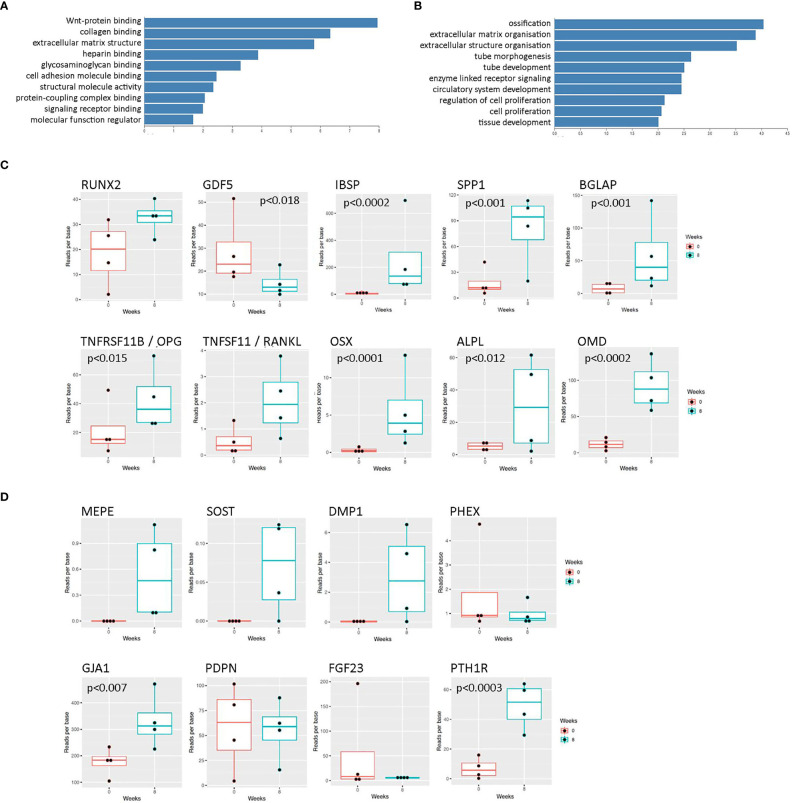
RNAseq data from human osteocyte constructs after 8 weeks of differentiation. **(A, B)** Graphs showing top over-represented pathways at 8 weeks from **(A)** KEGG pathway analysis and **(B)** GO terms in Webgestalt. Genes were considered differentially regulated based on an adjusted p value < 0.01. **(C, D)** Expression levels of selected transcripts at 0 weeks (red) and 8 weeks (blue) of differentiation related to **(C)** osteoblast differentiation and **(D)** osteocyte differentiation. Where p values are not shown, data is either not significant or p values are unavailable due to lack of detectable transcript at 0 weeks.

Initial rat osteocyte constructs used primary osteoprogenitor cells seeded into a fibrin/thrombin gel, which contracted around anchor posts fixed in the base of 6-well plates (19, 22). As a pilot study to determine whether the same methodology could be used to generate human osteocytes, gels were instead seeded with primary human osteoblasts from 4 different donors and the molecular signature of the cells within the constructs was assessed over 8 weeks (timeline, **Supplementary Figure 1**), focusing on markers of osteoblast and osteocyte differentiation and maturation (heat map; **Supplementary Figure 2**). This timeframe was estimated based on evidence of mineralization in rat constructs at 2-3 weeks (22). Encouragingly, at 8 weeks “Wnt signaling” was the top over-represented KEGG pathway (**Figure 1A**) and “ossification” was the first significant annotation using over-representation for GO terms in Webgestalt (**Figure 1B**). There was a general upregulation of specific markers of both osteoblast differentiation (**Figure 1C**; *RUNX2*, *TNFRSF11B* [*OPG*], *OSX, ALPL, OMB, IBSP, BGLAP, SPP1*) and osteocyte differentiation (**Figure 1D**; *DMP1, MEPE, GJA1, PTH1R, SOST*). Mineralization at 8 weeks was limited to focal spots scattered through the construct and around the hydroxyapatite post (uXRF; data not shown).

### 3.2 Human organotypic bone constructs contain viable cells in a mineralized matrix

**Figure 2:**
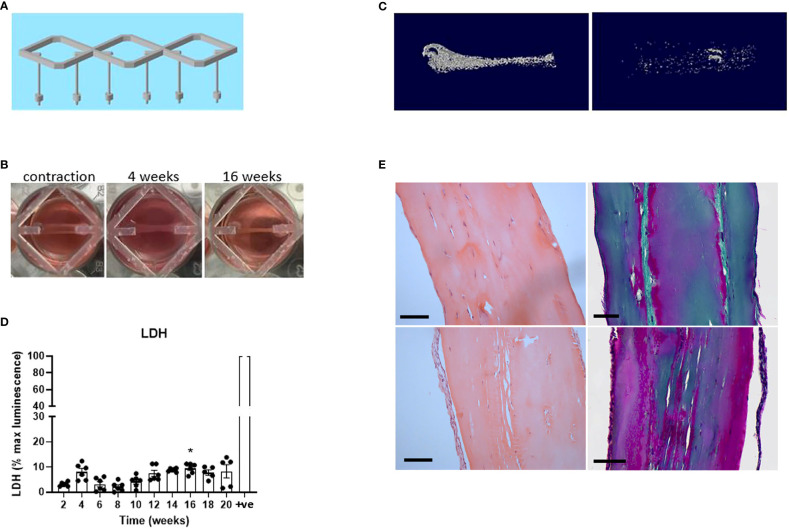
Human osteocyte constructs mineralize and are highly viable at 20 weeks. **(A)** Design of the 3D-printed scaffolds for use in 12-well dishes. **(B)** Human osteoblasts seeded into fibrin/thrombin gels contracted the gel between the posts and visibly thickened over time. **(C)** Representative reconstructed microCT images from two donors after 20 weeks of differentiation in osteogenic media. **(D)** Secreted LDH expressed as a percentage of the maximum LDH release control (+ve) obtained by treating constructs with 10% Triton-X100. **(E)** Histological staining of the constructs from panel **(C)** H&E staining (left panel); Masson’s trichrome stain (right panel), Scale bars = 100 μm. * p<0.05.

Although the pilot experiment suggested promising upregulation of early osteocyte markers, markers of mature osteocytes (e.g. *FGF23*) were not detected and mineralization was limited. Detection of new mineral was complicated by the calcium phosphate posts, which had been optimized for an earlier ligament model (23). It was therefore decided to increase the length of the differentiation period and to design 3D-printed scaffolds comprising triplicate hanging posts for 12-well dishes (**Figure 2A**). The new scaffolds would additionally reduce the scale of the model and increase flexibility (so that constructs could be moved between wells for e.g. co-culture experiments),

For this adapted model, primary human osteoblasts from six different donors were seeded into fibrin/thrombin gels which contracted between the hanging posts after approximately 2 weeks in proliferation media (timeline, **Supplementary Figure 1**). Osteogenic media was subsequently added twice weekly, resulting in visible further contraction (**Figure 2B**) and variable donor-dependent mineralization (**Figure 2C**) of the constructs by 20 weeks of osteogenic culture. Negligible cell death occurred over this timeframe as measured by secreted lactate dehydrogenase (LDH) assessed every 2 weeks (**Figure 2D**) and confirmed in 20-week constructs by live/dead staining (**Supplementary Figure 3**). Histological staining in the 20-week constructs revealed cells both aligned along the outer edge of the construct and buried in internal lacunae, with extensive production of collagen matrix aligned in the direction of the contractile force between the hanging posts (**Figure 2E**).

### 3.3 Human organotypic bone constructs contain mature osteocytes that secrete sclerostin and FGF23

**Figure 3:**
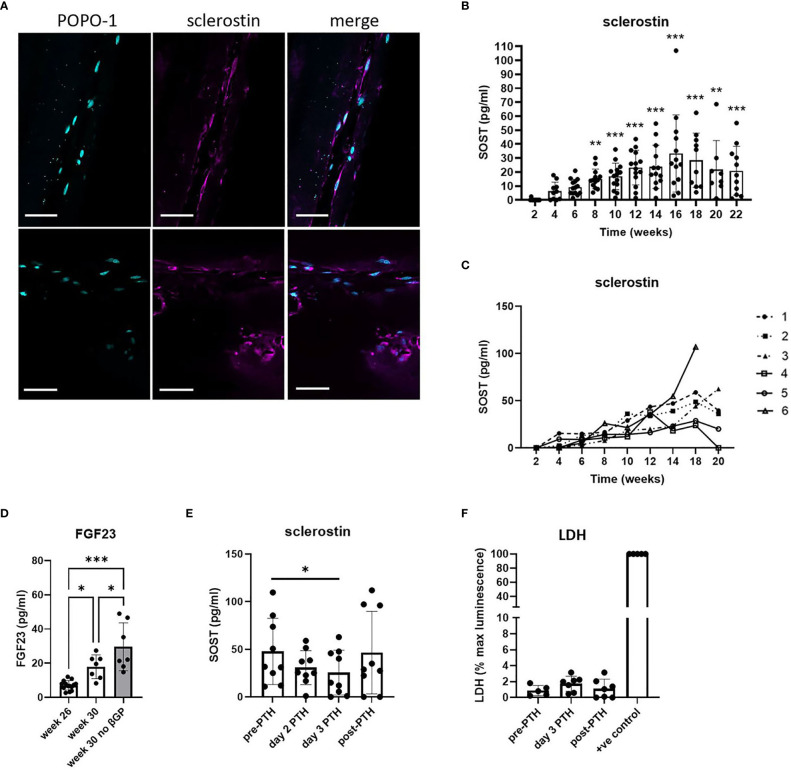
Human osteocyte constructs secrete sclerostin and FGF23. **(A)** Immunofluorescent staining of representative constructs for POPO-1 (blue) and sclerostin (purple). Scale bar = 50 μm. **(B, C)** Measurement of secreted sclerostin every 2 weeks shown **(B)** for all constructs from all 6 donors and **(C)** as a mean value per donor (donors numbered 1-6). **(D)** FGF23 secretion measured at 26 weeks and after 4 weeks further differentiation in osteogenic media in the presence or absence of β-glycerophosphate. **(E, F)** Constructs in the plateau phase of sclerostin secretion were assessed for **(E)** secretion of sclerostin and **(F)** LDH release immediately prior to treatment with 10nM PTH1-34 (pre-PTH), after 2 or 3 days of PTH1-34 treatment, and 7 days after removal of PTH1-34 (post-PTH). LDH is expressed as a percentage of the maximum LDH release control obtained by treating constructs with 10% Triton-X100 (+ve control). * p<0.05, ** p<0.01, *** p<0.001.

Cells within the 20-week bone constructs produced sclerostin protein, a marker of mature osteocytes. Most sclerostin-positive osteocytes were adjacent to the outer edge of the construct, aligned in the direction of the axial force, and displayed characteristic dendritic projections, although some were observed buried deeper within the tissue (**Figure 3A**; **Supplementary Figure 4**). Analysis of sclerostin secretion revealed an increase from 4 weeks of differentiation that reached significance at 8 weeks (p < 0.001) and plateaued from approximately 12 weeks (**Figure 3B**), indicative of the timecourse of differentiation of mature human osteocytes within the constructs. The high variability observed in the secretion of sclerostin was primarily due to high donor-to-donor variability in the response of the osteocytes generated from six different osteoblast donors, especially at later timepoints where sclerostin secretion by different donors diverged considerably (**Figure 3C**). Donor osteoblasts’ ability to form osteocytes in 3D (sclerostin secretion) did not correlate with characteristics in monolayer culture such as cell growth and osteogenic differentiation capacity (alkaline phosphatase, mineralization) (**Supplementary Figure 5**). However, the donor osteoblasts with very poor growth and osteogenic differentiation in monolayer culture did not efficiently contract the gel between the scaffold posts in 3D (**Supplementary Figure 5**).

FGF23, another secreted marker of mature osteocytes, was generally undetectable after 24 weeks of culture. However, constructs from two donors did secrete detectable FGF23 and, using only the FGF23-positive donors, FGF23 secretion was seen to increase further between 26 and 30 weeks of differentiation, indicative of continuing osteocyte differentiation and maturation during this period (**Figure 3D**). This increase was potentiated by removal of β-glycerophosphate from the differentiation media (**Figure 3D**). Removal of both β-glycerophosphate and dexamethasone (i.e. culture in proliferation media instead of osteogenic media) resulted in reduced metabolic activity and a reduction and/or delay in the secretion of mature osteocyte markers from the constructs (**Supplementary Figure 6**).

To assess whether sclerostin secretion by the human osteocyte constructs could be used to screen for osteocyte-targeting agents, constructs were exposed to the bone anabolic drug PTH1-34 (Teriparatide) which prevents osteocytic repression of bone formation by inhibiting sclerostin production (24). Treatment with 10 nM PTH1-34 reduced secretion of SOST1 (**Figure 3E**, 45.9% inhibition), secretion of which returned to baseline levels after removal of PTH1-34, without impacting cell viability (**Figure 3F**).

### 3.4 Vitamin D3 and hypoxia enhance mineralization and secretion of SOST1

**Figure 4:**
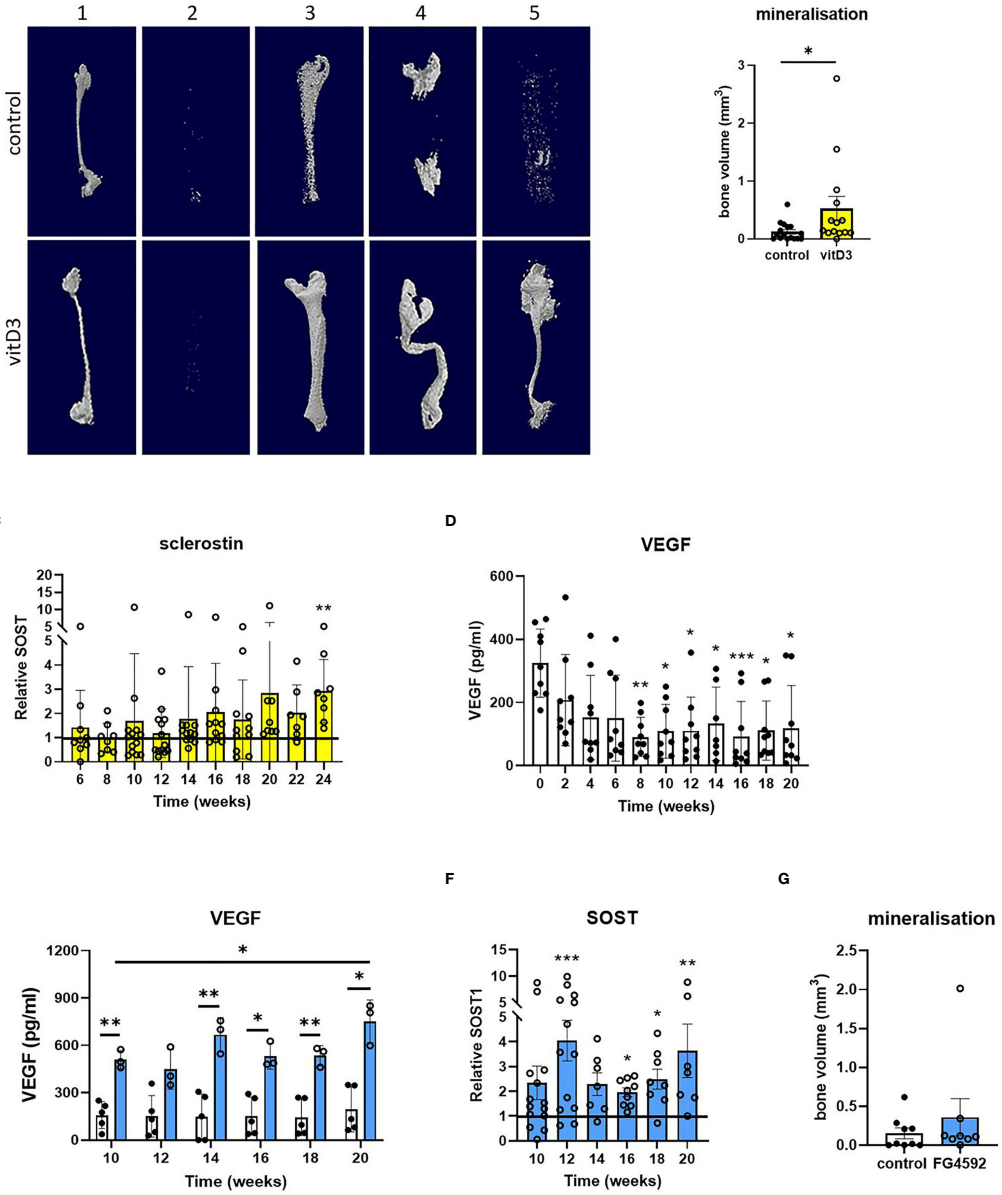
Effects of vitamin D3 and hypoxia on mineralization and secretion of SOST1. **(A)** Representative reconstructed microCT images and **(B)** quantified bone volume data for constructs from five osteoblast donors (numbered 1-5) after 20 weeks of differentiation in osteogenic media with 1 μM 25(OH)D3 (yellow bars) or without 25(OH)D3 (control/white bars). **(C)** Sclerostin secretion expressed as fold-change in 25(OH)D3-treated constructs relative to the unsupplemented (no 25(OH)D3) control. **(D, E)** VEGF secretion from osteocyte constructs **(D)** throughout the period of differentiation and **(E)** in response to supplementation with 5 μM FG4592 (blue bars). **(F)** Sclerostin secretion expressed as fold-change in FG4592-treated constructs relative to the unsupplemented (no FG4592) control. **(G)** Quantified bone volume data for constructs at 20 weeks of differentiation in osteogenic media with 5 μM FG4592 (blue bars) or without (white bars). *p<0.05, **p<0.01, ***p<0.001.

In an effort to improve the extent and reliability of mineralization, constructs were differentiated with the 25(OH)D3 metabolite of vitamin D3 which potentiates osteoblast differentiation and mineralization (25) (timeline, **Supplementary Figure 1**). Differentiation with 1 μM 25(OH)D3 increased osteoblast differentiation and mineralization in monolayer culture (**Supplementary Figure 7**) and increased mineralization in 3D osteocyte constructs from 4 out of 5 donors (**Figures 4A, B**). This was accompanied by a 1.4- to 5.2-fold increase in sclerostin secretion by 24 weeks of differentiation in 3D (**Figure 4C**, p<0.01), indicative of increased osteocyte activity.

As osteocytes are buried deep within mineralized bone, the hypoxic tissue microenvironment is central to osteocyte biology. Secretion of hypoxia-responsive vascular endothelial growth factor (VEGF) did not increase during the second half of the differentiation period during which mineralization occurs, despite an initial decrease when osteoblast proliferation was replaced by differentiation, suggesting that the constructs are not hypoxic (**Figure 4D**). The hypoxia-inducible factor (HIF) transcription factor regulates bone formation *in vivo* (26–28) and the HIF-prolyl hydroxylase inhibitor FG4592 (Roxadustat) stabilizes HIF-1α (29, 30). In primary human osteoblasts in monolayer culture, 5 μM FG4592 stabilized HIF-1α protein and increased expression of HIF-regulated Glut-1. However, the same dose inhibited osteoblast differentiation and mineralization (**Supplementary Figure 7**), as seen with other hypoxia mimetics (31). FG4592 was therefore only added to 3D osteocyte constructs from week 8 of differentiation, to avoid potential inhibitory effects on osteoblast differentiation (timeline, **Supplementary Figure 1**). Constructs treated with 5 μM FG4592 showed an immediate and sustained increase in secretion of both VEGF (**Figure 4E**) and sclerostin (**Figure 4F**), indicative of increased osteocyte activity, while avoiding inhibitory effects on mineralization (**Figure 4G**).

### 3.5 Co-culture with osteocyte constructs inhibits osteoclast differentiation

**Figure 5:**
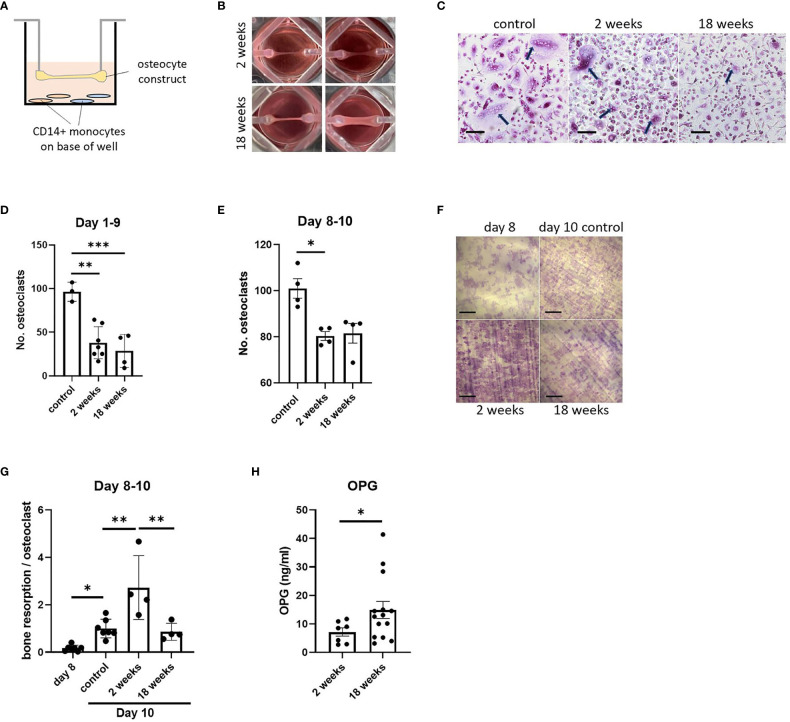
Effect of co-culture with osteocyte constructs on osteoclast differentiation and bone resorption. **(A)** Schematic showing co-culture of suspended osteocyte construct above CD14+ monocytes seeded on either glass coverslips (brown) or dentine discs (blue) on the base of the well. **(B)** Human osteocyte constructs are visibly thicker and more mineralized after 18 weeks in osteogenic media. **(C)** TRAP staining (arrows indicate osteoclasts, scale bar = 100 μm) and **(D)** quantification of the number of osteoclasts formed on coverslips after 9 days co-culture with 2-week or 18-week osteocyte constructs, or with a no-construct (osteoclast only) control, in the presence of M-CSF and RANKL. **(E–G)** Following 2 days co-culture of mature osteoclasts with 2-week or 18-week osteocyte constructs, or with a no-construct (osteoclast only) control, in the presence of M-CSF and RANKL; **(E)** quantification of the number of osteoclasts, **(F)** toluidine blue staining showing resorption pits on dentine discs (scale bar = 200 μm) and **(G)** quantification of the area of dentine resorbed per osteoclast. **(H)** OPG secretion measured at 2 weeks and 18 weeks of differentiation in osteogenic media, prior to co-culture experiments. *p<0.05, **p<0.01, ***p<0.001.

As well as regulating osteoblast function by secreting sclerostin, osteocytes regulate the formation and activity of osteoclasts via production of osteoclast-activating receptor activator of nuclear factor kappa-B ligand (RANKL) and the RANKL decoy receptor osteoprotegerin (OPG) (32). As a test of the flexibility of our osteocyte model in allowing co-culture with other cell types, constructs were co-cultured for up to 10 days suspended above primary human CD14+ monocytes that were induced to differentiate into osteoclasts by addition of exogenous macrophage colony stimulating factor (M-CSF) and RANKL (**Figure 5A**). Effects of co-culture with immature (2-week; containing mostly osteoblasts) and mature (18-week; containing mature osteocytes) constructs from matched donors (**Figure 5B**) were assessed on human osteoclast formation and bone resorption activity (timeline, **Supplementary Figure 1**). Co-culture with either 2-week or 18-week constructs inhibited osteoclast differentiation (**Figures 5C, D**). Acute co-culture of mature osteoclasts with either 2-week or 18-week osteocyte constructs for 2 days also resulted in fewer osteoclasts (**Figure 5E**); however, osteoclasts cultured with 2-week constructs had a 2.7-fold increase in bone resorption capacity (**Figures 5F, G**).

Such effects could be due to differences in the ratio of secreted RANKL : OPG from 2-week and 18-week constructs. In initial 8-week constructs, both mRNAs were detected with a RANKL : OPG ratio of 1:18 (**Figure 1B**). Analysis of secreted proteins revealed no detectable RANKL at any stage of differentiation (data not shown), suggesting that all expressed RANKL is in the membrane-bound form as in murine primary osteocytes (33). 18-week constructs secreted more OPG than 2-week constructs (**Figure 5H**), but this does not explain the increase in resorption during co-culture with 2-week constructs.

## 4 Discussion

This 3D organotypic model of human osteocyte formation demonstrates continuing sequential maturation of primary human osteocytes over a six-month period; mRNA for early osteocyte markers was present at 8 weeks, increasing secretion of sclerostin plateaued from 12 weeks, and FGF23 secretion increased from 24 weeks.

Osteocyte constructs displayed alignment of both matrix components and cell bodies in the direction of the contractile force that naturally arises between the hanging posts of the scaffolds (23). Grover et al. measured interface strength in the original ligament model using a custom-built tensile tester and the construct withstood load of up to 59 mN with the shape E posts used in our study. Mechanical stress caused failure at the interface at 9.5 kPa after 7 days in culture and around 20 kPa after 4 weeks (23). This contractile force, visible as a linear tightening of the constructs between the posts, is likely to expose the embedded cells to continuous moderate loading; this could represent a more physiological simulation of the mechanical environment experienced by osteocytes *in vivo* than standard monolayer or 3D models that are unloaded in their resting state (14, 34). Indeed, long-term mechanical loading is essential for osteocyte differentiation, lacunar-canalicular network formation, collagen I maturation and high organoid mineral density of human mesenchymal stem cells seeded on graphene oxide composite scaffolds (35). In addition to static load, constructs were removed from the incubator and fed twice weekly, providing a regular mechanical agitation that may also contribute to experienced load. Our constructs also have the advantage that they replicate the physiological spacing of osteocytes *in vivo*, with scattered cells in mineralized regions confined to their lacunae (1).

Although human osteocytes formed in the absence of osteogenic media, as reported in other 3D models (13), the differentiation process, assessed by secretion of sclerostin and FGF23, was significantly faster with osteogenic media. As demonstrated by the reversible anabolic repression of sclerostin by PTH1-34, our organoids allow screening for osteocyte-targeting agents via effects on secretion of sclerostin. It is therefore experimentally preferable to achieve measurable sclerostin levels as soon as possible and, for this reason, differentiation in osteogenic media was preferred with stable secretion of sclerostin from 12 weeks.

It is interesting to speculate whether the continuous moderate loading experienced in our model might be downregulating the amount of sclerostin secreted by the osteocyte constructs. Sclerostin mRNA and protein levels are dramatically reduced in response to loading *in vivo* (36), with areas of the bone receiving a greater mechanical strain intensity being associated with a greater reduction in both sclerostin staining intensity and percentage of sclerostin-positive osteocytes, with no positive cells in areas experiencing greater than 150με (37, 38). The same basic response occurs in primary human osteocytes *in vitro*; Sun et al. found that cyclic compressive pressure loading decreases SOST mRNA expression 30-fold in human primary osteocytes differentiated on calcium phosphate microbeads and reduces the percentage and intensity of staining of sclerostin-positive cells (14). It will be of interest to measure and adjust the strain intensity in our system in the future, to identify the effect of changes in strain intensity on sclerostin secretion over time.

Considerable inter-individual variation was evident between donors with respect to expression of osteocytic mRNAs, the extent of construct mineralization, and the amount of sclerostin and FGF23 that was secreted. Indeed, constructs failed from a small number of donors due to either insufficient ability to contract the gel between the posts or too great a contractility, in which case the constructs snapped (data not shown). Future development of this human 3D organoid model to compare the osteocyte response of bone cells taken from patients at different anatomical sites (e.g. with differing load-bearing) or of different age or gender will need to take such variation into account.

To potentially reduce this donor-to-donor variability, two physiological stimuli were assessed as methods of increasing osteocyte differentiation and activity; application of vitamin D3 and activation of hypoxia-regulated pathways. Serum concentrations of 25(OH)D3 strongly correlate with clinical parameters of bone health (39, 40). Bone cells convert 25(OH)D3 to 1,25(OH)2D3 using the 1α-hydroxylase enzyme CYP27B1 (41, 42). As well as promoting osteoblast mineralization (25), 1,25(OH)2D3 induces expression of key osteocyte genes including the mature osteocyte markers sclerostin and FGF23 (43, 44). In accordance with this literature, osteocyte constructs differentiated in the presence of 25(OH)D3 demonstrated enhanced mineralization and increased secretion of sclerostin, but without any apparent reduction in inter-individual variability in donor responses.

The oxygen tension of normal bone is 54.9–71.4 mmHg (6.6–8.6% O_2_) (45, 46). Osteoblasts reside on the bone surface adjacent to vascularized bone marrow, whereas osteocytes are buried in a relatively hypoxic microenvironment deep within mineralized bone. Hypoxia inhibits osteoblast differentiation and mineralization (47–49) but increases osteocyte differentiation and the expression and/or production of the mature osteocyte marker sclerostin, especially when either murine MC3T3-E1 cells (49) or primary human osteocytic cells (50) are grown in 3D culture. In our 3D organotypic culture system, delayed application of roxadustat caused a >3-fold increase in secretion of HIF-regulated VEGF and a 2- to 4-fold increase in secretion of sclerostin, supporting the ability of our model to mimic the known effects of hypoxia to increase osteocyte differentiation.

Dysregulation of FGF23 is linked with disorders of phosphate metabolism including hypophosphataemic rickets, chronic kidney disease and tumor-induced osteomalacia (3). Burosumab, an anti-FGF23 monoclonal antibody, has recently been approved as a therapeutic agent to treat these conditions (5). Osteocytes govern phosphate homeostasis through the production of FGF23, which lowers serum phosphate levels by increasing renal phosphate excretion and also inhibits mineralization (51, 52). The production of FGF23 in osteocytes is regulated by various local and systemic factors including phosphate; dietary phosphate loading increases serum FGF23 *in vivo* (53–55) and high phosphate increases FGF23 production *in vitro* (56). Our osteocyte constructs exhibited increasing secretion of FGF23 between 24 and 30 weeks of differentiation that was further elevated by removal of β-glycerophosphate from the osteogenic media. This finding is unexpected, however the regulation of FGF23 production is complex and multi-factorial (57). For example, Michigami et al. recently treated osteocytes derived from old and young mice with high phosphate and showed that, while FGF23 production increases in the osteocytes from the old mice, no effect is seen in those from the younger animals (56). The ability to manipulate production of FGF23 in our constructs enables future use of this organoid system for detailed molecular analysis of mechanisms associated with the bone-kidney interaction, including in co-culture studies.

Co-culture with CD14+ monocytes during monocyte-osteoclast differentiation was used as an illustration of the utility of the osteocyte organoid model in co-culture systems, with organoids and monocytes/osteoclasts being successfully maintained in co-culture for at least 10 days. Co-culture reduced osteoclast differentiation to 8.9-64.3% of control levels, with no difference observed in response between the immature and mature constructs. However, co-culture of mature osteoclasts with the immature constructs caused a 2.7-fold increase in bone resorption per osteoclast that was not observed during co-culture with the mature osteocyte constructs.

General inhibition of osteoclastogenesis could be due to the low ratio of RANKL : OPG evident at both the mRNA and protein level. Osteocytic RANKL is predominantly membrane-bound (33), meaning that osteocyte-monocyte co-culture can only promote osteoclast formation when cells are in direct contact in co-culture systems (33, 58, 59). Osteocyte-derived RANKL is a key contributor to disuse-induced bone loss, at least partially due to regulation of RANKL expression by mechanosensing and mechanotransduction (60). Using osteoblastic Saos-2 cells, for example, Galea et al. showed that exposure to strain by four-point bending downregulates expression of RANKL through a mechanosensitive epigenetic loop involving BRD2 (61). The continuous moderate loading experienced in our model might therefore contribute to the low RANKL : OPG ratio.

The physical separation of the cell types in our system resulted in effects of co-culture on osteoclast formation being dominated by high levels of secreted OPG. Unexpectedly, these high levels of OPG did not reduce bone resorption under co-culture conditions; bone resorption was even elevated by co-culture with immature constructs. This implies that osteocyte constructs might secrete a RANKL-independent factor that activates osteoclast bone resorption, but does not affect osteoclastogenesis, secretion of which is reduced as constructs mature (32, 62, 63); an avenue of research that will be of interest to pursue further.

In summary, we have developed a human 3D organotypic culture that replicates the processes of osteocyte burial in mineralizing osteoid through to the formation of mature osteocytes that secrete sclerostin and FGF23. Secreted sclerostin could be modulated reversibly by PTH1-34, demonstrating utility for bone anabolic drug discovery. Detectable FGF23 secretion will enable future development of a bone-kidney-parathyroid-vascular multi-organoid or organ-on-a-chip system to study disease processes and drug effects using purely human cells. This flexible, long-lived and versatile organotypic culture system provides a stable and regulated population of mature human primary osteocytes for a wide range of research applications.

## Data availability statement

The datasets presented in this study can be found in online repositories. The names of the repository/repositories and accession number(s) can be found below: https://www.ncbi.nlm.nih.gov/geo/query/acc.cgi?acc=GSE227994.

## Author contributions

Conception and design of the experiments was undertaken by PH, HK, LG, AC and APC. Collection, assembly, analysis, and interpretation of data was undertaken by PH, HK, AC, AK, and APC. The manuscript was prepared by HK and PH. All authors were involved in editing and approved the final version of the manuscript.

## References

[1] Plotkin LI Bellido T. Osteocytic signalling pathways as therapeutic targets for bone fragility. Nat Rev Endocrinol (2016) 12(10):593–605. doi: 10.1038/nrendo.2016.71 27230951 PMC6124897

[2] Balemans W Patel N Ebeling M Van Hul E Wuyts W Lacza C. Identification of a 52 kb deletion downstream of the SOST gene in patients with van buchem disease. J Med Genet (2002) 39(2):91–7. doi: 10.1136/jmg.39.2.91 PMC1735035 11836356

[3] Pathak JL Bravenboer N Klein-Nulend J. The osteocyte as the new discovery of therapeutic options in rare bone diseases. Front Endocrinol (2020) 11:405. doi: 10.3389/fendo.2020.00405 PMC7360678 32733380

[4] Marini F Giusti F Palmini G Brandi ML. Role of wnt signaling and sclerostin in bone and as therapeutic targets in skeletal disorders. Osteoporos Int (2023) 34(2):213–38. doi: 10.1007/s00198-022-06523-7 35982318

[5] Takashi Y Kawanami D Fukumoto S. FGF23 and hypophosphatemic Rickets/Osteomalacia. Curr Osteoporos Rep (2021) 19(6):669–75. doi: 10.1007/s11914-021-00709-4 34755323

[6] Wein MN Liang Y Goransson O Sundberg TB Wang J Williams EA. SIKs control osteocyte responses to parathyroid hormone. Nat Commun (2016) 7:13176. doi: 10.1038/ncomms13176 27759007 PMC5075806

[7] Dobrosak C Gooi JH. Increased sphingosine-1-phosphate production in response to osteocyte mechanotransduction. Bone Rep (2017) 7:114–20. doi: 10.1016/j.bonr.2017.10.002 PMC5651498 29085869

[8] Wang K Le L Chun BM Tiede-Lewis LM Shiflett LA Prideaux M. A novel osteogenic cell line that differentiates into GFP-tagged osteocytes and forms mineral with a bone-like lacunocanalicular structure. J Bone Miner Res (2019) 34(6):979–95. doi: 10.1002/jbmr.3720 PMC7350928 30882939

[9] Divieti Pajevic P. New and old osteocytic cell lines and 3D models. Curr Osteoporos Rep (2020) 18(5):551–8. doi: 10.1007/s11914-020-00613-3 32794140

[10] Zhang C Bakker AD Klein-Nulend J Bravenboer N. Studies on osteocytes in their 3D native matrix versus 2D *In Vitro* models. Curr Osteoporos Rep (2019) 17(4):207–16. doi: 10.1007/s11914-019-00521-1 PMC6647862 31240566

[11] Atkins GJ Welldon KJ Halbout P Findlay DM. Strontium ranelate treatment of human primary osteoblasts promotes an osteocyte-like phenotype while eliciting an osteoprotegerin response. Osteoporos Int (2009) 20(4):653–64. doi: 10.1007/s00198-008-0728-6 18763010

[12] Atkins GJ Welldon KJ Wijenayaka AR Bonewald LF Findlay DM. Vitamin K promotes mineralization, osteoblast-to-osteocyte transition, and an anticatabolic phenotype by gamma-carboxylation-dependent and -independent mechanisms. Am J Physiol Cell Physiol (2009) 297(6):C1358–67. doi: 10.1152/ajpcell.00216.2009 19675304

[13] Boukhechba F Balaguer T Michiels JF Ackermann K Quincey D Bouler JM. Human primary osteocyte differentiation in a 3D culture system. J Bone Miner Res (2009) 24(11):1927–35. doi: 10.1359/jbmr.090517 19419324

[14] Sun Q Choudhary S Mannion C Kissin Y Zilberberg J Lee WY. *Ex vivo* replication of phenotypic functions of osteocytes through biomimetic 3D bone tissue construction. Bone (2018) 106:148–55. doi: 10.1016/j.bone.2017.10.019 PMC5694355 29066313

[15] Brolese E Buser D Kuchler U Schaller B Gruber R. Human bone chips release of sclerostin and FGF-23 into the culture medium: an *in vitro* pilot study. Clin Oral Implants Res (2015) 26(10):1211–4. doi: 10.1111/clr.12432 24888411

[16] Pathak JL Bakker AD Luyten FP Verschueren P Lems WF Klein-Nulend J. Systemic inflammation affects human osteocyte-specific protein and cytokine expression. Calcif Tissue Int (2016) 98(6):596–608. doi: 10.1007/s00223-016-0116-8 26887974

[17] Mann V Huber C Kogianni G Jones D Noble B. The influence of mechanical stimulation on osteocyte apoptosis and bone viability in human trabecular bone. J Musculoskelet Neuronal Interact (2006) 6(4):408–17. PMC1847464 17185839

[18] Iordachescu A Hughes EAB Joseph S Hill EJ Grover LM Metcalfe AD. Trabecular bone organoids: a micron-scale 'humanised' prototype designed to study the effects of microgravity and degeneration. NPJ Microgravity (2021) 7(1):17. doi: 10.1038/s41526-021-00146-8 34021163 PMC8140135

[19] Iordachescu A Williams RL Hulley PA Grover LM. Organotypic culture of bone-like structures using composite ceramic-fibrin scaffolds. Curr Protoc Stem Cell Biol (2019) 48(1):e79. doi: 10.1002/cpsc.79 30644181

[20] Bolger AM Lohse M Usadel B. Trimmomatic: a flexible trimmer for illumina sequence data. Bioinformatics (2014) 30(15):2114–20. doi: 10.1093/bioinformatics/btu170 PMC4103590 24695404

[21] Bray NL Pimentel H Melsted P Pachter L. Near-optimal probabilistic RNA-seq quantification. Nat Biotechnol (2016) 34(5):525–7. doi: 10.1038/nbt.3519 27043002

[22] Iordachescu A Hulley P Grover LM. A novel method for the collection of nanoscopic vesicles from an organotypic culture model. RSC Adv (2018) 8(14):7622–32. doi: 10.1039/C7RA12511A PMC5819369 29568511

[23] Paxton JZ Donnelly K Keatch RP Baar K Grover LM. Factors affecting the longevity and strength in an *in vitro* model of the bone-ligament interface. Ann BioMed Eng (2010) 38(6):2155–66. doi: 10.1007/s10439-010-0044-0 PMC2871103 20431953

[24] Silva BC Costa AG Cusano NE Kousteni S Bilezikian JP. Catabolic and anabolic actions of parathyroid hormone on the skeleton. J Endocrinol Invest (2011) 34(10):801–10. doi: 10.3275/7925 PMC4315330 21946081

[25] Zarei A Hulley PA Sabokbar A Javaid MK Morovat A. 25-hydroxy- and 1alpha,25-dihydroxycholecalciferol have greater potencies than 25-hydroxy- and 1alpha,25-dihydroxyergocalciferol in modulating cultured human and mouse osteoblast activities. PLoS One (2016) 11(11):e0165462. doi: 10.1371/journal.pone.0165462 27893751 PMC5125576

[26] Wang Y Wan C Deng L Liu X Cao X Gilbert SR. The hypoxia-inducible factor alpha pathway couples angiogenesis to osteogenesis during skeletal development. J Clin Invest (2007) 117(6):1616–26. doi: 10.1172/JCI31581 PMC1878533 17549257

[27] Shomento SH Wan C Cao X Faugere MC Bouxsein ML Clemens TL. Hypoxia-inducible factors 1alpha and 2alpha exert both distinct and overlapping functions in long bone development. J Cell Biochem (2010) 109(1):196–204. doi: 10.1002/jcb.22396 19899108

[28] Qin Q Liu Y Yang Z Aimaijiang M Ma R Yang Y. Hypoxia-inducible factors signaling in osteogenesis and skeletal repair. Int J Mol Sci (2022) 23(19):11201. doi: 10.3390/ijms231911201 36232501 PMC9569554

[29] Dhillon S. Roxadustat: first global approval. Drugs (2019) 79(5):563–72. doi: 10.1007/s40265-019-01077-1 30805897

[30] Hulley PA Papadimitriou-Olivgeri I Knowles HJ. Osteoblast-osteoclast coculture amplifies inhibitory effects of FG-4592 on human osteoclastogenesis and reduces bone resorption. JBMR Plus (2020) 4(7):e10370. doi: 10.1002/jbm4.10370 32666021 PMC7340438

[31] Zahm AM Bucaro MA Srinivas V Shapiro IM Adams CS. Oxygen tension regulates preosteocyte maturation and mineralization. Bone (2008) 43(1):25–31. doi: 10.1016/j.bone.2008.03.010 18485858 PMC2504750

[32] Kitaura H Marahleh A Ohori F Noguchi T Shen WR Qi J. Osteocyte-related cytokines regulate osteoclast formation and bone resorption. Int J Mol Sci (2020) 21(14):5169. doi: 10.3390/ijms21145169 32708317 PMC7404053

[33] Honma M Ikebuchi Y Kariya Y Hayashi M Hayashi N Aoki S. RANKL subcellular trafficking and regulatory mechanisms in osteocytes. J Bone Miner Res (2013) 28(9):1936–49. doi: 10.1002/jbmr.1941 23529793

[34] Spatz JM Wein MN Gooi JH Qu Y Garr JL Liu S. The wnt inhibitor sclerostin is up-regulated by mechanical unloading in osteocytes *in vitro* . J Biol Chem (2015) 290(27):16744–58. doi: 10.1074/jbc.M114.628313 PMC4505423 25953900

[35] Zhang J Griesbach J Ganeyev M Zehnder AK Zeng P Schadli GN. Long-term mechanical loading is required for the formation of 3D bioprinted functional osteocyte bone organoids. Biofabrication (2022) 14(3). doi: 10.1088/1758-5090/ac73b9 35617929

[36] Zhao D Hua R Riquelme MA Cheng H Guda T Xu H. Osteocytes regulate bone anabolic response to mechanical loading in male mice via activation of integrin alpha5. Bone Res (2022) 10(1):49. doi: 10.1038/s41413-022-00222-z 35851577 PMC9293884

[37] Suzuki N Aoki K Marcian P Borak L Wakabayashi N. A threshold of mechanical strain intensity for the direct activation of osteoblast function exists in a murine maxilla loading model. Biomech Model Mechanobiol (2016) 15(5):1091–100. doi: 10.1007/s10237-015-0746-1 26578077

[38] Robling AG Niziolek PJ Baldridge LA Condon KW Allen MR Alam I. Mechanical stimulation of bone *in vivo* reduces osteocyte expression of sost/sclerostin. J Biol Chem (2008) 283(9):5866–75. doi: 10.1074/jbc.M705092200 18089564

[39] Bischoff-Ferrari HA Willett WC Wong JB Giovannucci E Dietrich T Dawson-Hughes B. Fracture prevention with vitamin d supplementation: a meta-analysis of randomized controlled trials. JAMA (2005) 293(18):2257–64. doi: 10.1001/jama.293.18.2257 15886381

[40] Need AG Horowitz M Morris HA Moore R Nordin C. Seasonal change in osteoid thickness and mineralization lag time in ambulant patients. J Bone Miner Res (2007) 22(5):757–61. doi: 10.1359/jbmr.070203 17280528

[41] Howard GA Turner RT Sherrard DJ Baylink DJ. Human bone cells in culture metabolize 25-hydroxyvitamin D3 to 1,25-dihydroxyvitamin D3 and 24,25-dihydroxyvitamin D3. J Biol Chem (1981) 256(15):7738–40. doi: 10.1016/S0021-9258(18)43337-6 6973569

[42] Geng S Zhou S Glowacki J. Effects of 25-hydroxyvitamin D(3) on proliferation and osteoblast differentiation of human marrow stromal cells require CYP27B1/1alpha-hydroxylase. J Bone Miner Res (2011) 26(5):1145–53. doi: 10.1002/jbmr.298 PMC3179303 21542014

[43] Wijenayaka AR Prideaux M Yang D Morris HA Findlay DM Anderson PH. Early response of the human SOST gene to stimulation by 1alpha,25-dihydroxyvitamin D(3). J Steroid Biochem Mol Biol (2016) 164:369–73. doi: 10.1016/j.jsbmb.2015.12.006 26690786

[44] Yashiro M Ohya M Mima T Nakashima Y Kawakami K Yamamoto S. Active vitamin d and vitamin d analogs stimulate fibroblast growth factor 23 production in osteocyte-like cells via the vitamin d receptor. J Pharm BioMed Anal (2020) 182:113139. doi: 10.1016/j.jpba.2020.113139 32045827

[45] Harrison JS Rameshwar P Chang V Bandari P. Oxygen saturation in the bone marrow of healthy volunteers. Blood. (2002) 99(1):394. doi: 10.1182/blood.V99.1.394 11783438

[46] Maurer P Meyer L Eckert AW Berginski M Schubert J. Measurement of oxygen partial pressure in the mandibular bone using a polarographic fine needle probe. Int J Oral Maxillofac Surg (2006) 35(3):231–6. doi: 10.1016/j.ijom.2005.07.016 16185845

[47] Salim A Nacamuli RP Morgan EF Giaccia AJ Longaker MT. Transient changes in oxygen tension inhibit osteogenic differentiation and Runx2 expression in osteoblasts. J Biol Chem (2004) 279(38):40007–16. doi: 10.1074/jbc.M403715200 15263007

[48] Utting JC Robins SP Brandao-Burch A Orriss IR Behar J Arnett TR. Hypoxia inhibits the growth, differentiation and bone-forming capacity of rat osteoblasts. Exp Cell Res (2006) 312(10):1693–702. doi: 10.1016/j.yexcr.2006.02.007 16529738

[49] Kim J Adachi T. Modulation of sost gene expression under hypoxia in three-dimensional scaffold-free osteocytic tissue. Tissue Eng Part A (2021) 27(15-16):1037–43. doi: 10.1089/ten.tea.2020.0228 33040693

[50] Choudhary S Sun Q Mannion C Kissin Y Zilberberg J Lee WY. Hypoxic three-dimensional cellular network construction replicates *ex vivo* the phenotype of primary human osteocytes. Tissue Eng Part A (2018) 24(5-6):458–68. doi: 10.1089/ten.tea.2017.0103 PMC5833258 28594289

[51] Sitara D Razzaque MS Hesse M Yoganathan S Taguchi T Erben RG. Homozygous ablation of fibroblast growth factor-23 results in hyperphosphatemia and impaired skeletogenesis, and reverses hypophosphatemia in phex-deficient mice. Matrix Biol (2004) 23(7):421–32. doi: 10.1016/j.matbio.2004.09.007 PMC2894977 15579309

[52] Nakatani T Sarraj B Ohnishi M Densmore MJ Taguchi T Goetz R. *In vivo* Genetic evidence for klotho-dependent, fibroblast growth factor 23 (Fgf23) -mediated regulation of systemic phosphate homeostasis. FASEB J (2009) 23(2):433–41. doi: 10.1096/fj.08-114397 PMC2630784 18835926

[53] Ferrari SL Bonjour JP Rizzoli R. Fibroblast growth factor-23 relationship to dietary phosphate and renal phosphate handling in healthy young men. J Clin Endocrinol Metab (2005) 90(3):1519–24. doi: 10.1210/jc.2004-1039 15613425

[54] Perwad F Azam N Zhang MY Yamashita T Tenenhouse HS Portale AA. Dietary and serum phosphorus regulate fibroblast growth factor 23 expression and 1,25-dihydroxyvitamin d metabolism in mice. Endocrinology (2005) 146(12):5358–64. doi: 10.1210/en.2005-0777 16123154

[55] Takashi Y Kosako H Sawatsubashi S Kinoshita Y Ito N Tsoumpra MK. Activation of unliganded FGF receptor by extracellular phosphate potentiates proteolytic protection of FGF23 by its O-glycosylation. Proc Natl Acad Sci USA. (2019) 116(23):11418–27. doi: 10.1073/pnas.1815166116 PMC6561303 31097591

[56] Michigami T Tachikawa K Yamazaki M Nakanishi T Kawai M Ozono K. Growth-related skeletal changes and alterations in phosphate metabolism. Bone (2022) 161:116430. doi: 10.1016/j.bone.2022.116430 35577326

[57] Michigami T. Roles of osteocytes in phosphate metabolism. Front Endocrinol (2022) 13:967774. doi: 10.3389/fendo.2022.967774 PMC9334555 35909535

[58] Bernhardt A Osterreich V Gelinsky M. Three-dimensional Co-culture of primary human osteocytes and mature human osteoclasts in collagen gels. Tissue Eng Part A (2020) 26(11-12):647–55. doi: 10.1089/ten.tea.2019.0085 31774039

[59] Bernhardt A Skottke J von Witzleben M Gelinsky M. Triple culture of primary human osteoblasts, osteoclasts and osteocytes as an *In Vitro* bone model. Int J Mol Sci (2021) 22(14):7316. doi: 10.3390/ijms22147316 34298935 PMC8307867

[60] Sasaki F Hayashi M Ono T Nakashima T. The regulation of RANKL by mechanical force. J Bone Miner Metab (2021) 39(1):34–44. doi: 10.1007/s00774-020-01145-7 32889574

[61] Galea GL Paradise CR Meakin LB Camilleri ET Taipaleenmaki H Stein GS. Mechanical strain-mediated reduction in RANKL expression is associated with RUNX2 and BRD2. Gene (2020) 763S:100027. doi: 10.1016/j.gene.2020.100027 34493364

[62] Knowles HJ Cleton-Jansen AM Korsching E Athanasou NA. Hypoxia-inducible factor regulates osteoclast-mediated bone resorption: role of angiopoietin-like 4. FASEB J (2010) 24(12):4648–59. doi: 10.1096/fj.10-162230 PMC2992372 20667978

[63] Knowles HJ Athanasou NA. Canonical and non-canonical pathways of osteoclast formation. Histol Histopathol (2009) 24(3):337–46. doi: 10.14670/HH-24.337 19130404

