# Supplementary material for: Mature primary human osteocytes in mini organotypic cultures secrete FGF23 and PTH1-34-regulated sclerostin
Source: Front Endocrinol (Lausanne). 2023 May 8;14:1167734. doi: 10.3389/fendo.2023.1167734 (PMC10200954; doi:10.3389/fendo.2023.1167734)
Supplement: Supplementary file 1 [file DataSheet_1.docx]

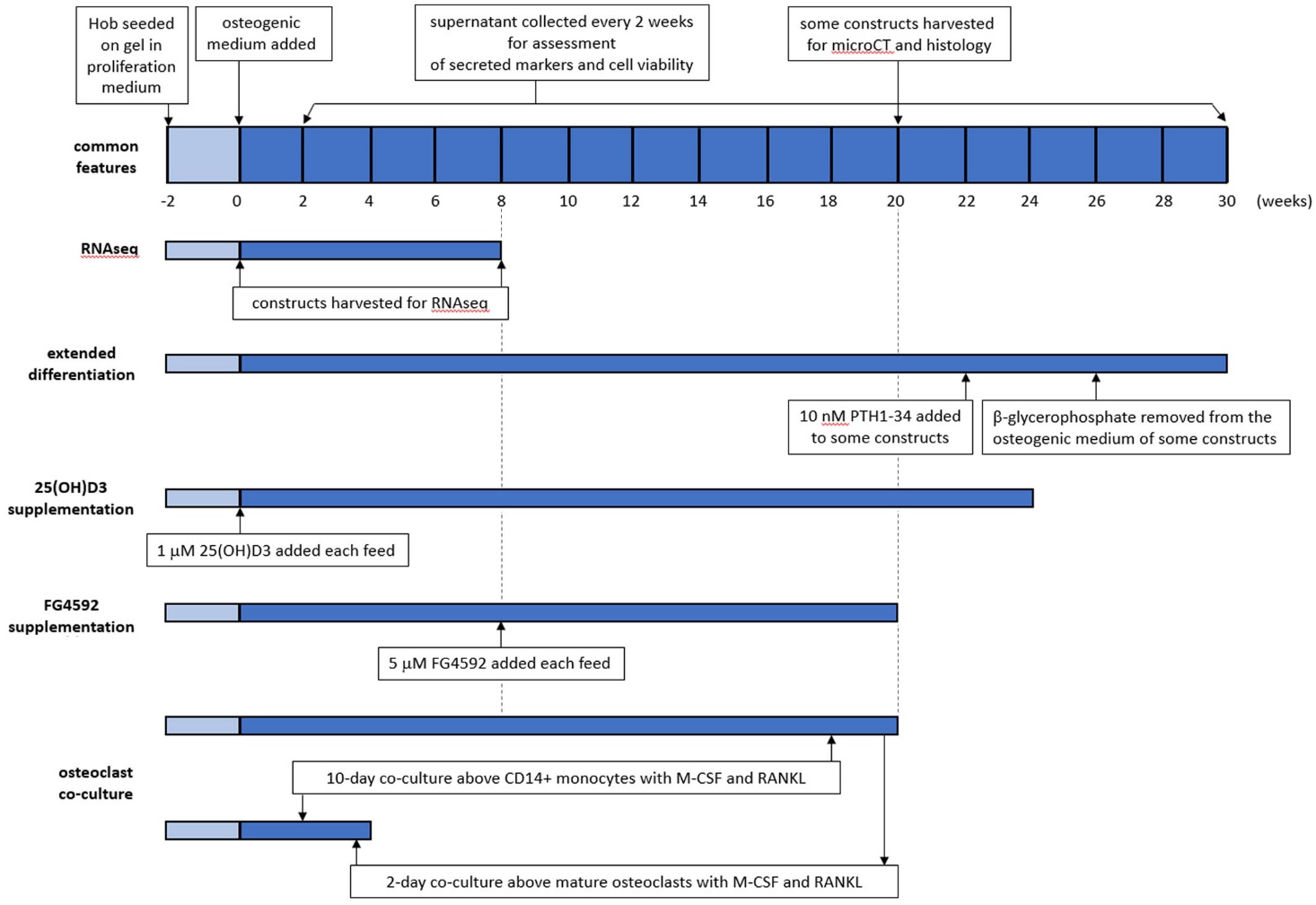


**Supplementary Figure 1: Timeline of osteocyte construct experiments.** Schematic showing common features of osteocyte construct experiments (top bar) as well as individual features relevant to specific named experiment sets.


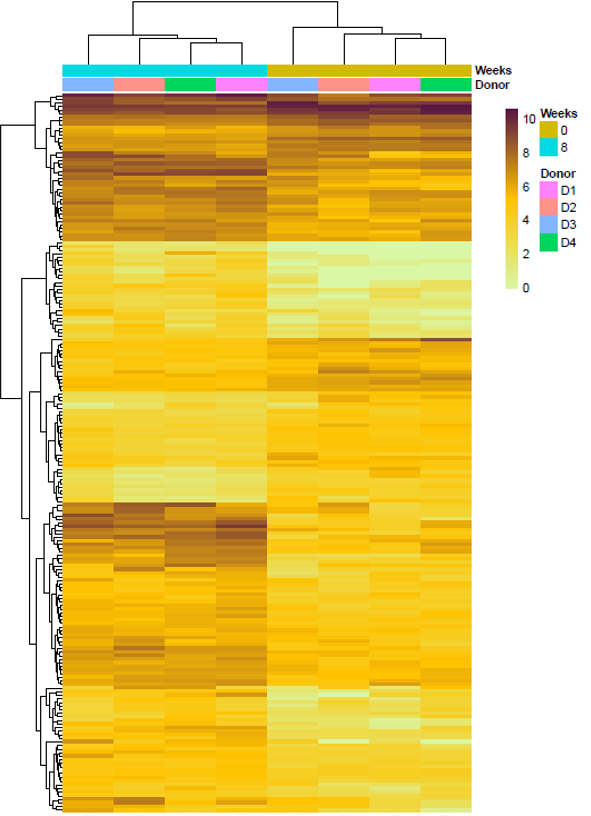


**Supplementary Figure 2:** **RNAseq data from human osteocyte constructs after 8 weeks of differentiation.** Heat map generated in R using ggplot2 showing relative gene expression in constructs seeded with osteoblasts from 4 donors (D1-D4) at 0 weeks (gold, right hand side) and 8 weeks (blue, left hand side) of differentiation. Genes were considered differentially regulated based on an adjusted p value < 0.01.


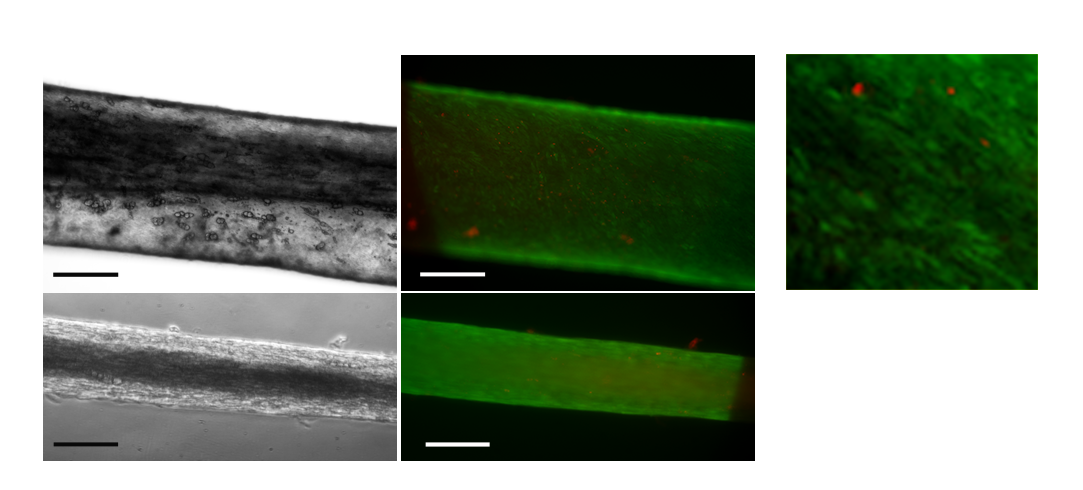


**Supplementary Figure 3: Human osteocyte constructs are highly viable at 20 weeks.** Phase contrast image (left) and live / dead staining (middle) of representative 20-week constructs. Right: magnification of boxed area in middle image. Green = live (calcein-AM); red = dead (ethidium homodimer-1); scale bar = 400 μm.


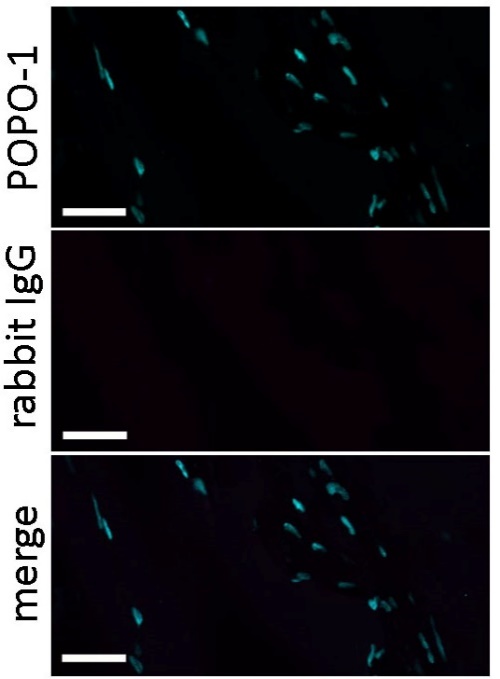


**Supplementary Figure 4: Human osteocyte constructs secrete sclerostin.** Negative control for sclerostin immunofluorescence using rabbit IgG; POPO-1 (blue) and rabbit IgG (purple). Scale bar = 50 μm.


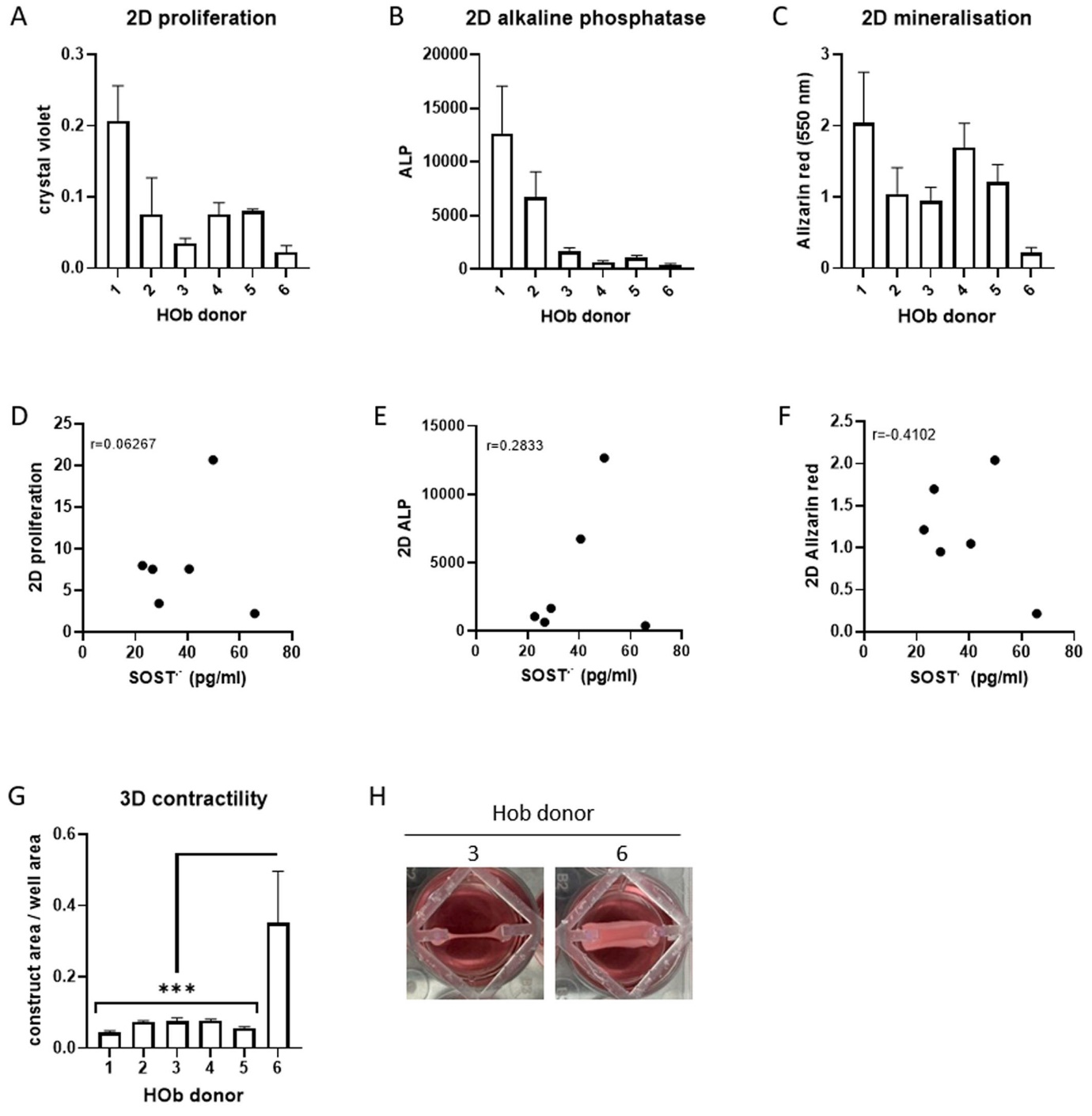


**Supplementary Figure 5: Donor cell characteristics in monolayer culture do not predict osteocyte formation in 3D culture.** Donor-specific quantification of (A) cell proliferation, (B) early osteogenic differentiation and (C) mineralisation in monolayer culture (D-F) did not correlate with the mean amount of sclerostin secreted per donor during the plateau phase (week 12-16) of secretion in 3D culture; (D) cell proliferation vs sclerostin, (E) early osteogenic differentiation vs sclerostin, (F) mineralisation vs sclerostin. (G) Gel contractility at 16 weeks measured as area of osteocyte construct / area of well. (H) Representative images of constructs from donor 3 and donor 6 in 12-well plates. *** p<0.001.


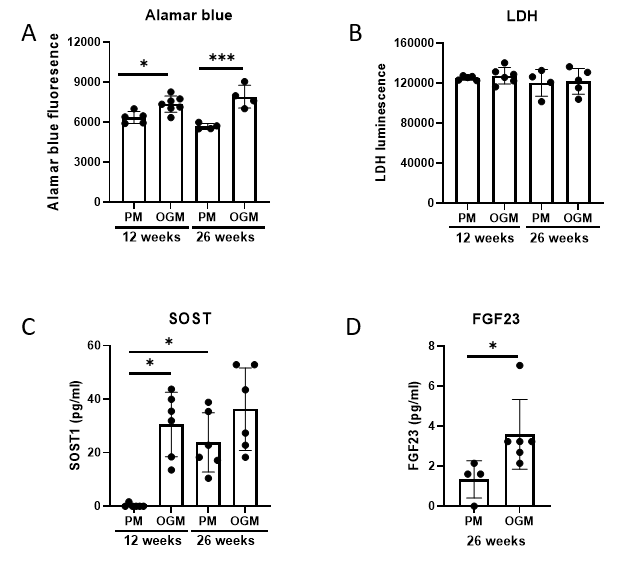


**Supplementary Figure 6: Human organotypic bone constructs form mature osteocytes in the absence of osteogenic media.** Comparison of osteocyte constructs differentiated in proliferation media (PM) or osteogenic media (OGM) at 12 and 26 weeks of differentiation: (A) cell metabolic activity (alamar blue fluorescence); (B) cell death (LDH release); (C) secretion of sclerostin; (D) FGF23 secretion. * p<0.05, ** p<0.01, *** p<0.001.


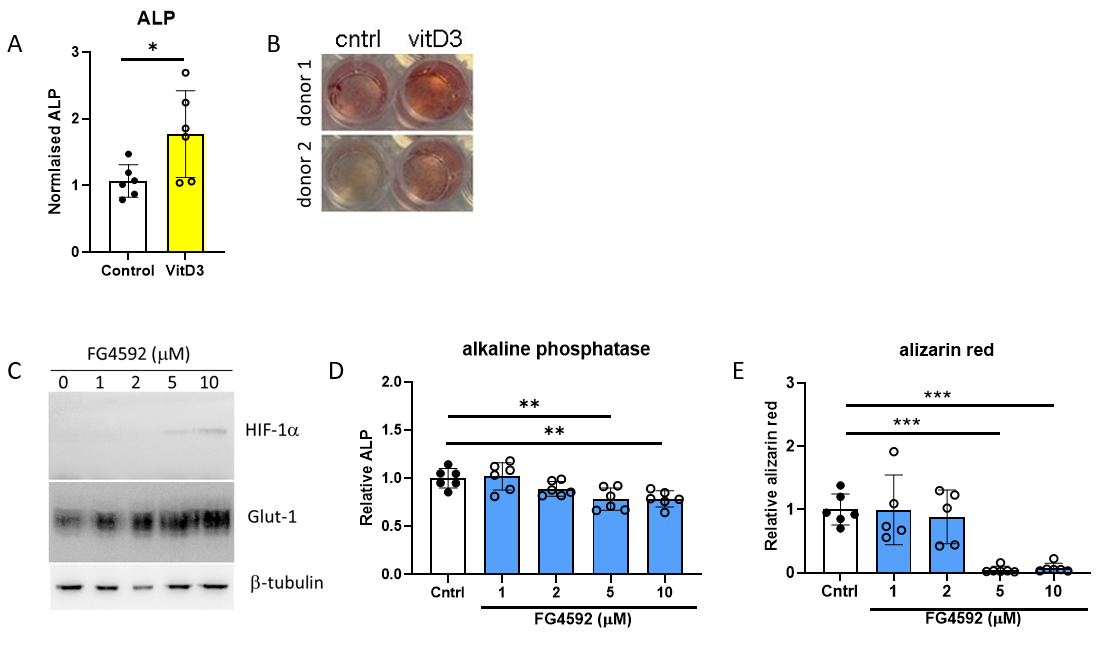


**Supplementary Figure 7: Effect of vitamin D3 and FG4592 on osteoblast differentiation in monolayer culture.** (A, B) Differentiation of primary human osteoblasts with osteogenic media supplemented with 1 μM 25(OH)D3; (A) alkaline phosphatase (ALP) activity and (B) mineralisation (alizarin red staining). (C) Western blot following exposure of primary human osteoblasts to 1-10 μM FG4592 for 16h. (D, E) Differentiation of primary human osteoblasts with osteogenic media supplemented with 1-10 μM FG4592; (A) alkaline phosphatase activity and (B) mineralisation (alizarin red staining). * p<0.05, ** p<0.01, *** p<0.001.
